# A rare case of sigmoid diverticulitis presenting as colocutaneous left lumbar fistula

**DOI:** 10.1093/jscr/rjae487

**Published:** 2024-08-06

**Authors:** Vesna Potkonjak, Petar Milic, Ljiljana Vuckovic, Jelena Perunovic, Dario Potkonjak

**Affiliations:** Department of Surgery, General Hospital Kotor, Kotor 85330, Montenegro; Department of Surgery, General Hospital Kotor, Kotor 85330, Montenegro; Department of Radiology, General Hospital Kotor, Kotor 85330, Montenegro; Clinic for Pathology and Citology, Clinical Center of Montenegro, Podgorica 81100, Montenegro; Clinic for Digestive surgery, University Clinical Center of Serbia, Belgrade 11000, Serbia

**Keywords:** sigmoid diverticulitis, lumbar fistula, colocutaneous fistula

## Abstract

Colonic diverticula are very common as asymptomatic findings on endoscopic examination. Diverticulitis as a complication occurs in ~4% of these patients with well-known further complications. Colocutaneous fistulas are very rare and are usually caused by percutaneous drainage procedures of abscess formations or as a complication of the natural disease. We present here a case report of a 70-year-old man who initially presented with signs of sepsis and later developed a colocutaneous fistula in the lumbar region.

## Introduction

Colonic diverticula are routinely discovered as asymptomatic findings during endoscopic examination in a large proportion of the population, with a prevalence of around 65% in patients over 65 years of age. The prevalence is increasing as a result of Western lifestyles, such as low-fiber diets, alcohol consumption, sedentary lifestyles, and the aging of the population. Diverticulitis is a complication of diverticular disease that occurs in ~4% of patients. The most common complications of diverticulitis are bleeding, perforation, abscesses, and fistula formation.

Colocutaneous fistulas are very rare, accounting for 1%–4% of fistula formation after acute diverticulitis. Much more common are colovesical and colovaginal fistulas, which manifest as pneumaturia, fecaluria, dysuria, and recurrent urinary tract infections. Fistulas may be the result of percutaneous drainage of abscess colic during acute Hinchey 1b or II diverticulitis or postoperatively after colonic resection with primary anastomosis [1]. Much less frequently, they can form spontaneously as the first symptom of the disease. We report on a patient with acute diverticulitis presenting as abscess formation with colocutaneous fistula in the lumbar region.

## Case report

A 74-year-old male patient was admitted as an emergency in severe general condition with signs of septic shock and severe pain in the lumbar region and fever (38.7°C). Physical examination revealed a phlegmon in the left lumbar region. The patient had a history of uncomplicated diverticular disease with three previous attacks in the last 8 years. Elective surgical treatment was previously suggested according to the guidelines, but the patient refused it.

Blood work revealed a high white blood cell count of 35.8 × 109/L with a left shift and a C-reactive protein (CRP) of 292 mg/L.

An urgent multidetector computed tomography of the abdomen and pelvis with contrast medium (Fig. 2) confirmed an abscess accumulation in the retroperitoneal space with diverticular disease of the left colon, and also in the lumbar region a large inflammatory accumulation with gas inclusions with a diameter of 120 × 70 mm, as well as spread to the iliac compartment and inflammation of the psoas muscle.

After a brief preoperative preparation, the patient was taken to the operating room as an emergency to make a surgical incision and drain the abscess. Perioperatively, a complex abscess was found, which produced a large amount of pus after drainage. On the second postoperative day, the drainage tube began to fill with gaseous feces, which continued for three more days. After 8 days, there was no further discharge and no more gas, only minimal serous secretion.

**Figure 1:**
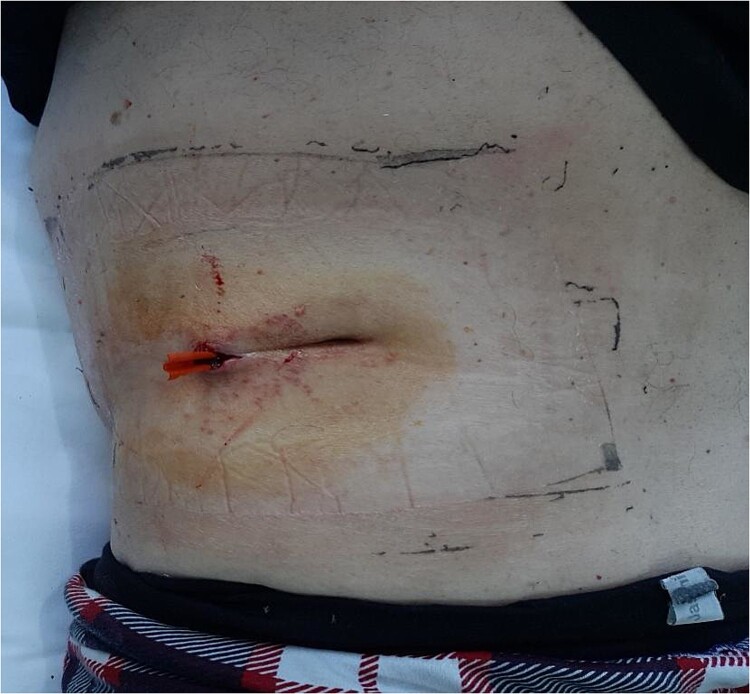
Site of lumbar incision with drain.

After a prolonged course of antibiotics, which according to the antibiogram included third generation cephalosporins in combination with metronidazole, the abdominal symptoms and sepsis improved over the following 17 days. There was a gradual recovery and a good regression of the abscess formation. We continued to monitor the patient’s condition. The secretion at the previous incision site and drainage persisted and required daily dressing changes (Fig. 1).

**Figure 2:**
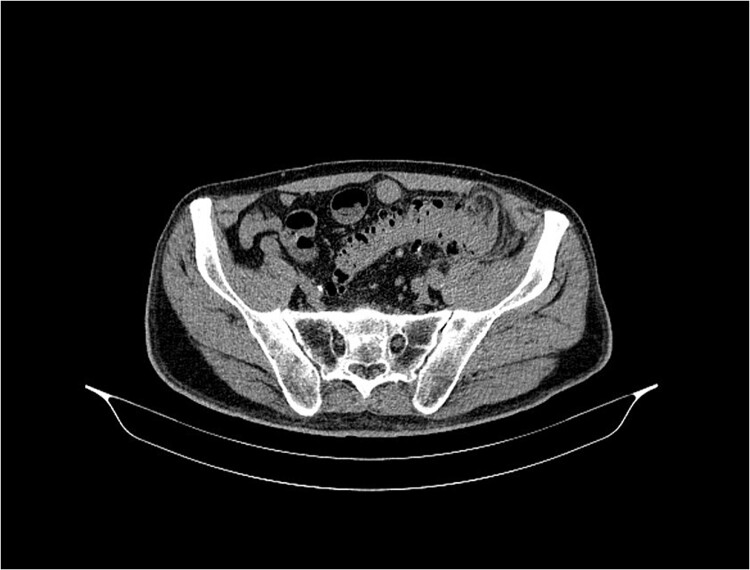
MSCT of the abdomen and pelvis shows the sigmoid colon and skin communication with air accumulation in the axial plane.

The control MDCT showed a condition after percutaneous drainage of a previously verified larger abscess collection in the iliopsoas region, where post-inflammatory inhomogeneous zones with a diameter of 25 mm were now seen, without clearly developed collections, but with a fistulous tract between the bowel and the left lumbar region (Fig. 2a and b). The patient was discharged from the hospital in good condition. Laboratory white blood cell count and inflammatory parameters were normal (WBC 9.5 × 109/L and CRP 2.7 mg/L).

Although we recommended surgical treatment, the patient declined this for the next 10 months while continuing clinical outpatient clinic monitoring and dressing changes, with minimal discharge around the surgical incision.

During follow-up, a colonoscopy was performed, which showed massive diverticulosis of the descending and sigmoid colon, with clear demarcation of a larger diverticulum in the distal part of the descending colon, with edematous and hyperemic mucosa.

Twelve months after the initial presentation, the patient was readmitted to the hospital due to a septic condition and recurrent phlegmon in the lumbar region. The laboratory parameters were high with a white blood cell count of 16 × 109/L and a CRP level of 176 mg/L).

MDCT scan of the abdomen and pelvis showed marked diverticulosis of the sigmoid and descending colon with dense fluid accumulation in the ileopsoas region and fistula formation in the lumbar region. Limited collections of thick fluid in the abdominal wall of the lumbar region with a diameter of 56 × 57 mm were also detected. The findings in the abdominal cavity were normal, with no presence of free fluid.

Dual antibiotic therapy was administered with renewed drainage of the lumbar region. Ten days later, after the general condition had improved and the inflammatory process had subsided, which was confirmed by control laboratory analyses and a control echo examination, elective surgical treatment was now proposed and confirmed by the patient.

**Figure 3:**
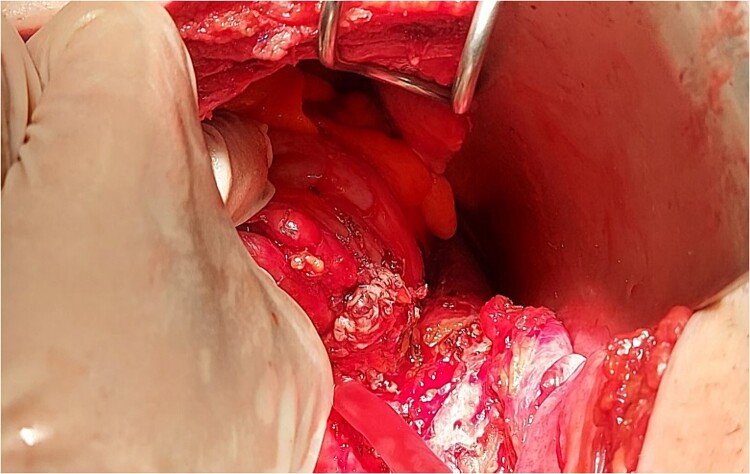
Intraoperative findings of fistula tract.

**Figure 4:**
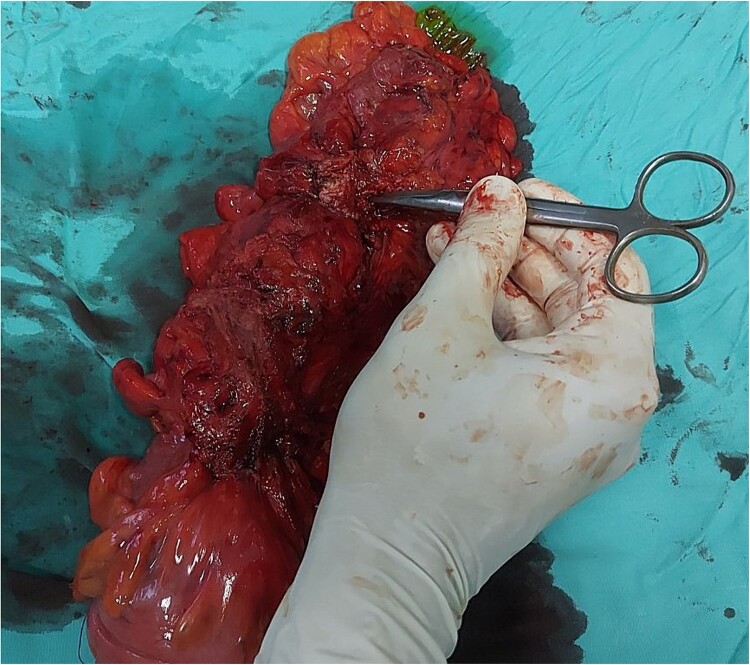
Specimen with marked place of fistula.

**Figure 5:**
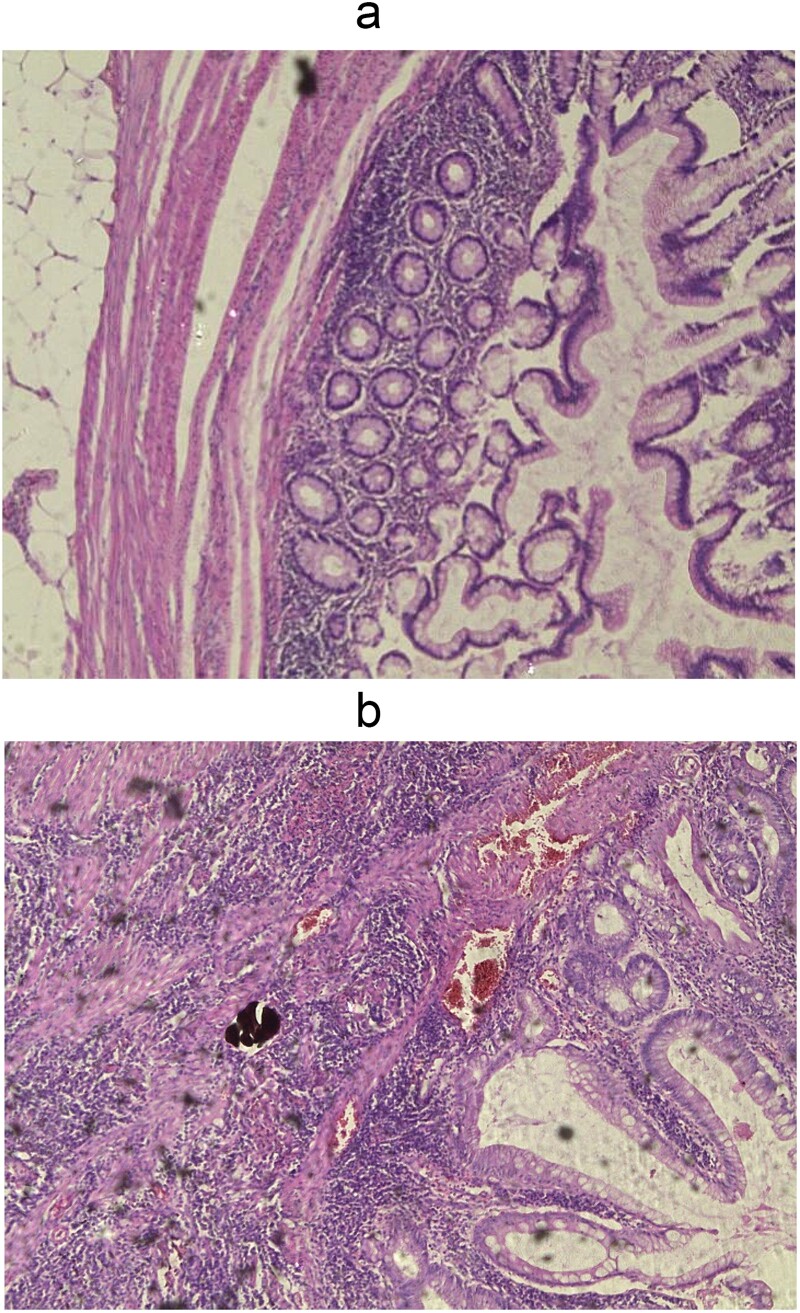
Wall of diverticulum in fat tissue (a) and acute purulent inflammation of diverticulum (b), H&E, 5x enlargement.

The intraoperative findings (Fig. 3) and the specimen (Fig. 4) show a spot in the sigmoid colon communicating with the left lumbar region. We performed a segmental resection and restored bowel continuity using a double stapling technique. We also placed a drain at the site of the previous lumbar incision. Pathologic examination of the resected colon revealed sigmoid diverticulitis and no histologic evidence of Crohn’s disease or actinomycosis (Fig. 5a and b).

## Discussion

Diverticula are sac-shaped protrusions of the colon wall. They can be false diverticula, which affect the mucous membrane and the muscularis mucosae, or true diverticula, in which all layers of the intestinal wall are affected.

About 95% of diverticular diseases originate in the sigmoid and left colon (95%). These are false diverticula, which are much more common in the western world. In contrast, in the East, diverticula are more common on the right side of the colon, where they are true diverticula.

Diverticulitis is one of the most common inflammatory diseases of the gastrointestinal tract. A century ago, this disease was uncommon, but today it leads to around 300 000 hospital admissions per year. This makes it one of the five most costly diseases of the gastrointestinal tract in the United States [2]. Readmission rates are also high, with a 90-day readmission rate of ~8.9%, with additional costs and a mortality rate of ~5% [3].

Diverticulosis of the colon covers a broad clinical spectrum. The presentation can range from asymptomatic patients (see below) to complicated diverticulitis. Although the exact percentage of asymptomatic patients is not known, it is believed that the majority of patients remain asymptomatic. This group of patients is diagnosed incidentally during screening colonoscopy for colorectal cancer or for other reasons [4].

In the group of patients who develop symptoms, a quarter are referred for bleeding, and the remaining three quarters develop signs of diverticulitis [5].

Approximately a quarter of patients with diverticulitis develop acute complications, such as rectal bleeding or perforation, as well as chronic complications, such as bowel strictures or fistulas, with adjacent organs and structures [6].

Disease-specific classifications have been developed to try to predict the outcome of diverticulitis. In 1978, Hinchey classification was developed at that time for the evaluation of perforated diverticulitis [7]. In later decades, some modifications were developed to differentiate not only complicated diverticulitis but also mild disease. There is a clinically relevant correlation between disease stage and mortality risk. Several studies have documented mortality rates of 5–10 for patients with diverticulitis and abscess formation (Hinchey I/II) and 20%–50% for patients with perforated disease with peritonitis (Hinchey III/IV) [8–10].

Colocutaneous fistulas are very rare, accounting for 1%–4% of the total number of fistulas as a complication of diverticular disease and can be caused by percutaneous drainage of a diverticular abscess [11]. They occur more frequently after primary resection and anastomosis.

To our knowledge, only a few cases of sigmoid diverticulitis presenting as flank abscess have been reported.

Charalabopoulos *et al.* described a similar case of an 85-year-old woman with a subcutaneous abscess on the left flank.

Bakopoulos *et al.* had a rare case of diverticulitis of the transverse colon that manifested with a colocutaneous fistula.

An abscess originating from diverticulitis of the colon can mimic other serious conditions.

Patrick Bates Murphy and Paul Belliveau reported a case of left-sided diverticulitis presenting as a right-sided thigh abscess. The case of Alice Rubartelli *et al.* showed that perforated colonic diverticulitis can cause fasciitis of the limbs.

The most important diagnostic method for acute diverticulitis is multidetector CT with intravenous contrast medium. MDCT offers a sensitivity of 98% and a specificity of up to 99% for the diagnosis of acute diverticulitis [12].

The medical history can help in making the correct diagnosis.

On admission, patients with AD usually present with signs of sepsis, abdominal pain, fever, malaise, and nausea. In this scenario, the first and most important goal is aggressive resuscitation with control of the source (preferably by percutaneous drainage), after which a fistula may develop. After full recovery, definitive surgery is planned.

It is clear that in recent decades there has been a trend toward laparoscopic sigmoidectomy for the treatment of both uncomplicated and complicated diverticulitis. It is interesting to note that most of these studies generally excluded complicated cases, such as abscesses or fistulas. There is little data in the English literature for complicated cases, and surgery certainly requires more expertise in these cases. Laparoscopic colectomy has replaced open resection as the standard operation for recurrent and complicated diverticulitis in many institutions [13].

Three randomized trials have shown that minimally invasive surgery (laparoscopic, robotic) for diverticular disease is associated with lower short-term morbidity. Management of diverticulitis morbidity in terms of less blood loss, less postoperative pain, and shorter hospital stay compared to open surgery [14–16]. It would be important to make a clear distinction between emergency surgery for damage limitation and elective surgery for persistent or recurrent disease. Unfortunately, many studies in the literature either refer only to the latter or do not provide the necessary transparency of data. Long-term outcomes are comparable, with an overall improvement in satisfaction with the minimally invasive approach [17, 18].

Although diverticular disease is a benign process, it can sometimes pose significant technical challenges in surgery due to the chronic inflammatory process or after initial treatment leading to distortion of the anatomy. In such a situation, the use of hand-assisted laparoscopic surgery (HALS) with tactile sensation could have some benefit. There are two small randomized trials comparing laparoscopic surgery with HALS surgery for diverticular disease. They reported shorter overall operating times but a slightly longer length of stay (1 day longer for HALS surgery). No significant difference was found in short-term postoperative morbidity [6, 7].

To the best of our knowledge, the approach to emergency surgery for complicated diverticulitis with abscess and fistula is damage limitation based on the patient’s condition and comorbidities. In our case, we chose to address the septic condition with an incision and abscess drainage rather than resection, which would result in prolonged operative time, complications, and potentially unfavorable outcomes for the patient.

At our institution, we have not yet started performing laparoscopic sigmoidectomies in patients with complicated diverticulitis and fistula, so we have chosen the open approach due to inexperience with these cases and patients.

Similar case reports have been published.

## Conclusions

Colocutaneous fistula is a rare complication of the disease but should not be overlooked by the clinician. In patients with the clinical manifestation of a subcutaneous abscess in the left lumbar region and previous episodes of diverticulitis that have been treated conservatively, sigmoid perforation and the formation of an abdominal wall abscess, as in our case, should be suspected.

The decision on the correct treatment of this clinical condition should be based on a thorough clinical examination and imaging.

## Data Availability

All data is available in the hospital records of the patient.
